# *Avibacterium paragallinarum*: Pathogenesis Mechanisms and Subunit Vaccine Development

**DOI:** 10.3390/microorganisms14051093

**Published:** 2026-05-12

**Authors:** Zhihua Li, Ying Liu, Zhenyi Liu, Zhaoling Jiang, Yawen Wang, Baozhu Xing, Chen Mei, Hongjun Wang

**Affiliations:** Institute of Animal Husbandry and Veterinary Medicine, Beijing Academy of Agriculture and Forestry Sciences, Beijing 100097, China

**Keywords:** *A. paragallinarum*, tissue tropism, pathogenic mechanism, subunit vaccine, mucosal immunity

## Abstract

*Avibacterium paragallinarum* (*A. paragallinarum*) is the primary causative agent of infectious coryza in chickens. Infection often leads to growth retardation in broilers and a 10% reduction in egg production, reaching over 40% in laying hens. The problem is particularly severe under intensive farming conditions, significantly jeopardizing global poultry health and farming profitability. From a ‘One Health’ perspective, this not only disrupts the stability of the food supply chain, but also increases antibiotic usage due to disease prevention and control needs, thereby aggravating antimicrobial resistance (AMR) and posing a global public health challenge. This review systematically summarizes advances in the pathogenesis of *A. paragallinarum* and the protective immunity induced by subunit vaccines. It focuses on the infection mechanisms of *A. paragallinarum*, emphasizing its colonization strategies in the infraorbital sinus and nasal epithelium of chickens, and analyzes the roles of key virulence factors such as hemagglutinin and capsule in adhesion, colonization, and immune evasion. We integrate the tissue-specific pathogenesis of *A. paragallinarum* with the role of respiratory commensal microbiota in facilitating infection, providing an in-depth analysis of the bacterium’s key immune evasion strategies, thus offering novel insights into host–pathogen-microbiome interactions. Concurrently, to the best of our knowledge, this review provides the first comprehensive overview of current developments in subunit vaccines and their immunoprotective properties, with special attention to limitations in eliciting mucosal immune responses. By delving into the pathogen-host interaction mechanisms, this review aims to inform the optimization of subunit vaccine design and immunization strategies. Ultimately, it seeks to establish a theoretical basis and practical framework for precise control of *A. paragallinarum*.

## 1. Introduction

*A. paragallinarum* is the primary causative agent of infectious coryza in chickens, an acute upper respiratory disease manifested by symptoms such as nasal discharge, facial swelling, and conjunctivitis [1]. Infection frequently results in growth retardation in broilers and a significant decline in egg production by 10% to over 40% in laying hens. The economic impact is particularly severe under intensive farming conditions. Furthermore, the pathogenicity of *A. paragallinarum* is often complicated and exacerbated by concurrent infections, which substantially compromises poultry health and farm productivity [2,3,4]. This disease has a global distribution, with reported in major poultry-producing regions including the United States, China, Brazil, the United Kingdom, and South Africa [5,6].

**Figure 1:**
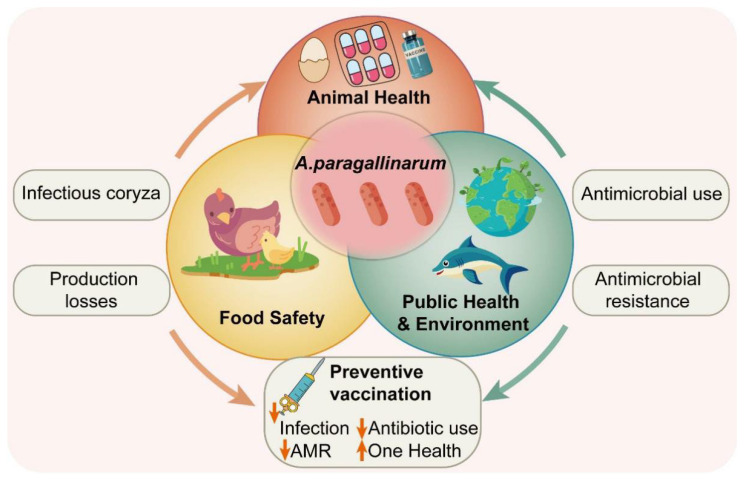
One Health perspective of *A. paragallinarum* Infection. This schematic represents the *A. paragallinarum* infection through the lens of the One Health approach, illustrating the dynamic interactions among animal health, food safety, public health, and environmental sustainability. It highlights the consequences of infectious coryza in poultry, including its detrimental effects on animal welfare and productivity, the reliance on antimicrobial interventions, and the associated risk of AMR. These interconnected factors may not only threaten food security and safety but also pose broader implications for ecosystem health and One Health resilience.

In recent years, the significance of *A. paragallinarum* extends beyond poultry production. Within the “One Health” framework, the bacterium has the potential to affect animal, environmental, and public health. Evidence confirms its cross-species transmission within wildlife populations, where it can infect endangered avian species such as the Golden Pheasant (*Chrysolophus pictus*) and the Reeves’s Pheasant (*Syrmaticus reevesii*), causing lethal epidemics [7]. This not only threatens biodiversity but also risks establishing wildlife reservoirs for novel variants, complicating disease control efforts. Moreover, the extensive use of antimicrobials in farming has contributed to the increasing severity of AMR in pathogens including *A. paragallinarum* [8]. This trend may cause treatment failures, economic losses, and public health risks due to drug residues in poultry products (Figure 1) [9,10].

Currently, various inactivated and subunit vaccines are available for prevention. However, the protective efficacy of these vaccines—particularly against heterologous serotypes and emerging variant strains—remains uncertain under field conditions. This is due to limited cross-protection among serotypes and the continuous emergence of variant or endemic strains [11]. Therefore, unraveling the pathogenic mechanisms and tissue specificity of *A. paragallinarum*, as well as addressing the challenges in vaccine development, is urgently needed. Substantial research has been devoted to elucidating its infection mechanisms. The bacterium shows marked tissue tropism during natural infection. Pure cultures can typically be recovered from the deep infraorbital sinuses of infected chickens using sterile swabs [12]. Additionally, infiltration by inflammatory cells, macrophages, and heterophils is confined to the nasal cavity and infraorbital sinuses in infected chickens [13]. These observations imply the existence of specific tissue adherence mechanisms in niches such as the infraorbital sinuses. Understanding the pathogenic mechanism is essential to develop a vaccine against it. Subunit vaccines against *A. paragallinarum* are predominantly administered via intramuscular or subcutaneous injection. While effective in eliciting systemic humoral immunity, they can also prevent local mucosal infection. In fact, these upper respiratory sites rely on mucosal immunity—which comprises secretory IgA and mucosa-associated lymphoid tissue—as the first line of defense against respiratory pathogens. However, the mechanisms underlying this protection are not fully elucidated [14,15,16]. Consequently, investigating the mechanisms of tissue-selective infection and local immune protection from the perspective of host–pathogen interactions will be instrumental. This research can help develop more targeted vaccines, optimize immunization strategies, and advance novel vaccines with enhanced efficacy and broader cross-protective coverage.

This review systematically summarizes recent advances in the pathogenic mechanisms of *A. paragallinarum*, with a focus on its key virulence determinants, tissue-specific infection strategies and the immune protection conferred by subunit vaccines. It seeks to provide a theoretical foundation to improve targeted prevention and control strategies against this pathogen.

## 2. Biological Characteristics and Pathogenicity of *A. paragallinarum*

### 2.1. Taxonomic Characteristics and Serotyping

*A. paragallinarum*, a member of the family Pasteurellaceae, is a Gram-negative, non-motile, non-spore-forming bacterium that possesses a capsule and exhibiting bipolar staining. Its morphology is characterized as short rods or coccobacilli. The majority of strains require an exogenous supply of NAD^+^ (V factor) for growth [1,17,18,19]. However, NAD-independent strains have been identified and reported in regions such as Mexico and South Africa [1,20,21]. These traits are closely associated with its capacity for colonization, survival, and pathogenesis within the host.

Currently, serotyping mainly uses two schemes: the Page and Kume schemes. The Page scheme classifies *A. paragallinarum* into three serovars: A, B and C. In contrast, the Kume scheme categorizes them into three serogroups: A, B and C, which are further subdivided into nine serotypes (A-1, A-2, A-3, A-4, B-1, C-1, C-2, C-3, and C-4) [22,23]. The hemagglutinin gene *HMTp210* is recognized as the primary molecular determinant of serotype. Region 2 of this gene is hypervariable, and its sequence variation correlates with serotype specificity [24,25]. Further research has identified that variations within the first 1200 nucleotides of the *HMTp210* gene coding sequence strongly correlate with serotype classification. This region corresponds to approximately the first 400 amino acids at the protein’s N-terminus. The region harbors multiple copies of the Hep_Hag domain, which is composed of 7-amino-acid repeat sequences. Based on the co-localization of its sequence features with serotype determinants, these Hep_Hag domains are hypothesized to play a critical role in mediating the binding of the HMTp210 protein to chicken erythrocytes, potentially governing the serogroup specificity of the hemagglutinin [23]. Epidemiological investigations have revealed variations in the distribution of *A. paragallinarum* serotypes across different geographical regions and time periods. For instance, isolates from Taiwan, China (1994–2017) were predominantly serogroups B and C [23], whereas the predominant serotypes identified in Central China (2019–2022) were A-1 and C-4 [26]. These differences likely result from factors including geography, environmental, and vaccination practices [27]. Virulence can vary significantly not only among different serotypes but also among distinct strains within the same serotype, with some strains exhibiting heightened pathogenicity [12,28]. Notably, serogroups A, B, and C represent three distinct immunotypes, and vaccine-induced protection demonstrates marked serotype specificity [29]. This is because sequence variations in *HMTp210* region 2 across serotypes lead to antigenic differences. Antibodies induced by one serotype often fail to effectively neutralize heterologous serotypes, directly explaining the limited cross-protection and the need for multivalent vaccines [28]. Consequently, vaccine design must account for both serotype diversity and prevailing epidemiological characteristics to enhance its efficacy and specificity.

### 2.2. Infection Process and Clinical Manifestations

Following infection with *A. paragallinarum*, affected chickens often exhibit lethargy and reduced intake of feed and water. Clinical signs primarily include facial swelling centered on the infraorbital sinuses, nasal discharge, sneezing, and increased ocular secretions [30]. Inflammatory exudate can spread downward within subcutaneous spaces due to gravity, leading to inflammation and swelling in the mandibular space and wattles. The exudate can also enter the oral cavity and trachea via the posterior nasal cleft, causing tracheitis. Further progression to the lower respiratory tract can result in airsacculitis, pneumonia, and other lower respiratory tract lesions, potentially developing into systemic infection. Although infectious coryza is generally confined to the upper respiratory system with a low incidence of systemic infection [31], studies have isolated the pathogen from non-respiratory organs, suggesting the possibility of systemic dissemination [32,33]. Davison et al. investigated an infectious coryza outbreak in Pennsylvania, USA (2018–2019) and found that *A. paragallinarum* infection was not limited to the upper respiratory tract. The pathogen was isolated from organs such as the lungs, air sacs, heart, and liver in some layers and broilers [30]. In an experimental infection study, Guo et al. detected high levels of the pathogen not only in primary target organs like the nasal cavity and infraorbital sinuses but also in systemic sites including blood, lungs, and spleen. The bacterial load in the blood peaked on day 5 post-infection (9.16 × 10^5^ CFU/g), demonstrating that *A. paragallinarum* can breach the local mucosal barrier, enter the bloodstream, and cause bacteremia [13]. It should be noted that the field outbreak reported by Davison et al. involved frequent viral and bacterial co-infections, and the experimental study by Guo et al. used a relatively high challenge dose (10^7^ CFU/mL, 0.2 mL per chicken) administered via the infraorbital sinus, a more severe route than natural infection. Collectively, these findings indicate that under specific conditions, the pathogen is capable of systemic dissemination. This phenomenon may be associated with bacterial co-infection, strain variation, environmental stress, or the route and dose of infection.

### 2.3. Pathogenic Factors

The pathogenicity of *A. paragallinarum* is a multifactorial process, in which various virulence factors act synergistically to mediate key steps such as bacterial adhesion, colonization, invasion, and immune evasion. Hemagglutinin (HA) is a key virulence factor and immunogen of *A. paragallinarum*, playing a central role in bacterial adhesion, colonization, and the elicitation of immune responses. Early studies demonstrated that strains carrying the HA antigen could be reisolated from chickens for a period post-infection, whereas strains lacking this antigen were rapidly cleared by the host, suggesting that HA mediates initial adhesion or colonization [34]. Wang et al. identified the HMTp210 protein as the major HA of *A. paragallinarum*. Their study showed that the *HMTp210* gene deletion mutant completely lost hemagglutination activity, failed to induce hemagglutination-inhibition (HI) antibodies, and exhibited significantly reduced adhesion to HeLa cells as well as impaired biofilm formation. Animal challenge experiments also demonstrated attenuated virulence [35]. This suggests that HMTp210 mediates the initial adhesion of bacteria, promotes biofilm formation, and underlies a potential mechanism for immune evasion and persistent infection. Additionally, the HMTp210 protein is a trimeric autotransporter adhesin (TAA). TAAs are an important class of bacterial surface virulence factors known to mediate the initial adhesion to host cells and the extracellular matrix, promote bacterial aggregation and biofilm formation, and enhance bacterial resistance to host immune clearance and antibiotic action. Many TAAs directly participate in immune evasion by binding to complement regulatory proteins (such as C4BP and factor H) or immunoglobulins in serum, thereby inhibiting complement activation pathways and phagocytosis, and conferring serum resistance to the bacteria [16,35,36,37]. In addition to its role as a virulence factor, HA also serves as an important protective immunogen capable of inducing high titers of HI antibodies and is a key antigen for subunit vaccines. This vaccine-related aspect will be discussed in detail in Section 5.

The capsule is another core factor determining the pathogenicity of *A. paragallinarum*. Sawata and Kume demonstrated that smooth colony variants with abundant capsules were highly virulent, whereas rough variants lacking capsules were avirulent. Although hyaluronic acid, a major component of the capsule, can mask the underlying specific HA antigen and affect initial bacterial adhesion and colonization [34], Tu et al. found that the capsule is a key virulence factor in resisting host immune clearance, protecting bacteria from phagocytosis and complement-mediated killing. They constructed a mutant by knocking out the capsular polysaccharide transporter gene *hctA*. Even though this mutant exhibited enhanced hemagglutination and adhesion activity, its virulence was significantly attenuated in animal challenge experiments, confirming that the primary role of the capsule lies in immune evasion rather than in promoting adhesion [38]. This simultaneously indicated that *hctA* is a virulence gene in *A. paragallinarum*. Shelkamy et al., while comparing the genomes of pathogenic and non-pathogenic *A. paragallinarum*, discovered that the *hctA* gene was exclusive to pathogenic strains [38,39,40]. This suggests that the virulence of *A. paragallinarum* depends not only on adhesion but also on the ability to survive in the host and evade immune clearance. Moreover, the critical role of the capsule has been corroborated by recent studies focused on vaccine development. Strains with intact capsules serving as vaccine candidates can induce more effective immune protection, suggesting that capsular antigens are ideal targets for subunit vaccine development [41,42].

In addition to hemagglutinin and the capsule, *A. paragallinarum* expresses a variety of other virulence factors that collectively promote its infection and pathogenic processes. Pili are important surface structures mediating initial bacterial adhesion. Liu et al. identified the F17-like pilus protein FlfA in *A. paragallinarum* and confirmed its role in virulence through gene knockout and animal challenge experiments. Compared to the wild-type strain, the *flfA* knockout mutant caused significantly lower clinical symptom scores and markedly attenuated virulence. Immunization of chickens with recombinant FlfA protein alleviated clinical symptoms post-challenge. This evidence indicates that FlfA plays a key role as both a virulence factor and a potential vaccine antigen [14].

Iron is a limiting nutrient for pathogen survival within the host. Huo et al. revealed that the heme utilization protein HutZ plays a crucial role in iron homeostasis and pathogenicity of *A. paragallinarum*. The HutZ mutant exhibited restricted growth in medium with heme as the sole iron source and a reduced tolerance to acidic conditions, indicating the protein’s importance in heme degradation, iron release, and adaptation to host environmental stress. In terms of pathogenicity, HutZ deficiency impaired the ability of *A. paragallinarum* to invade and survive within the chicken macrophage cell line HD11. In vivo experiments further showed that the mutant caused reduced morbidity and milder clinical symptoms [43]. These finding establish HutZ as an important virulence factor and a potential target for subunit vaccine development.

Glutathione reductase (GR) is a key enzyme for maintaining bacterial redox homeostasis, and its role in the pathogenicity of *A. paragallinarum* has been confirmed. Studies have shown that loss of GR function severely disrupts the endogenous redox balance, leading to accumulation of reactive oxygen species (ROS), depletion of reduced glutathione (GSH), and disruption of NAD(H)/NADP(H) metabolism. This intrinsic oxidative stress state compromises bacterial viability, resulting in a significant decline in growth rate. Furthermore, GR deficiency attenuates bacterial virulence. The mutant exhibits severely impaired biofilm formation, significantly reduced adhesion to and invasion of HD11 chicken macrophages, and attenuated virulence in specific-pathogen-free (SPF) chickens. These results indicate that while GR is not a virulence factor that directly attacks the host, it plays a supporting role in pathogenicity by regulating the bacterial oxidative stress response and metabolic energy balance, thereby providing the necessary intracellular environment for the expression and function of other virulence factors [44].

## 3. Pathogenic Mechanisms of *A. paragallinarum*

### 3.1. Experimental Observations on Tissue Specificity

**Figure 2:**
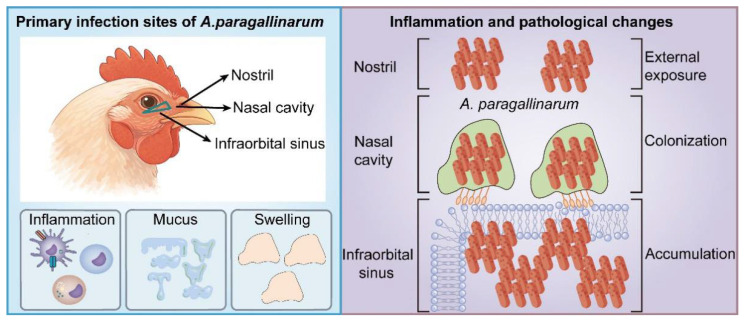
Tissue tropism and infection process of *A. paragallinarum*. Following exposure, *A. paragallinarum* preferentially colonizes the nasal cavity, infraorbital sinuses, trachea, and upper respiratory tract of chickens. The infection induces mucosal inflammation, mucus accumulation, and impaired respiratory function-hallmarks of infectious coryza. This tissue-specific pathogenesis underpins the clinical manifestations and transmission dynamics of the disease within poultry populations.

Tissue specificity is a central feature in the pathogenesis of *A. paragallinarum*. Both experimental and field observations consistently indicate that the bacterium exhibits a tropism for the upper respiratory tract of chickens, particularly the infraorbital sinuses and nasal mucosa (Figure 2). Davison et al. reported isolation of the bacterium from the infraorbital sinuses in 36.6% of cases, second only to the choanal cleft (56.6%). Necropsy findings further indicated that swollen infraorbital sinuses with exudate were the primary lesion, with a detection rate of 100% in broilers and pullet flocks [30]. Lu et al. immunized SPF chickens with the capsule-hemoprotein via either intramuscular injection in the pectoral muscle or infraorbital sinus injection, followed by a challenge through the infraorbital sinus. The results showed that immunization via the infraorbital sinus induced lower serum antibody titers compared to pectoral muscle injection, yet conferred higher protection (80% vs. 60%) [45]. This discrepancy may be related to the tissue-specificity of the infection.

Additionally, Guo et al. found that bacterial loads in the nasal cavity continuously increased until the late stage of infection, whereas in tissues such as the lung, spleen, and trachea, they initially increased and then declined. This pattern suggests that *A. paragallinarum* can undergo transient widespread dissemination during the early infection stage, but ultimately establishes persistent colonization primarily in the upper respiratory tract, especially the nasal cavity [13]. Immunohistochemical analysis by Balouria et al. revealed that during the early infection stage, *A. paragallinarum* specifically adhered to and colonized the keratinized epithelium of anterior nasal turbinates and the respiratory epithelium of middle turbinates in chickens. Bacterial antigens were distributed as granular deposits in the superficial epithelial layers. As the infection progressed, bacterial antigens appeared in the infraorbital sinus, triggering a host innate immune response. Macrophages phagocytosing bacterial antigens were present in the subepithelial region, accompanied by infiltration of IgA^+^ lymphocytes and monocytes at sites of bacterial colonization and migration [46]. This phenomenon reveals early interactions between the pathogen and the local immune system.

In summary, multiple studies have established a clear tissue predilection of *A. paragallinarum* for the upper respiratory tract, particularly the infraorbital sinuses and nasal cavity. Notably, this tropism has been consistently observed across different strains, including Pennsylvania field isolates (serotype C) [30], strain BJ-05 (serovar B) [45], strain 2020/JS80 (serovar C) [13], and a serovar B isolate [46], suggesting it is a conserved species characteristic rather than strictly strain-dependent. This tissue-specific colonization pattern implies the existence of molecular interactions between bacterial surface components and host epithelial receptors at these sites.

### 3.2. Potential Binding Targets in Infection: Pathogen Adhesins and Host Receptors

The critical initial step in the pathogenesis of *A. paragallinarum* is its adhesion to and colonization of the host respiratory mucosa [36]. Studies indicate that the hemagglutinin protein HMTp210 is involved in initial bacterial attachment to host cells and is classified as a trimeric autotransporter adhesin (TAA) [35]. TAAs are important virulence factors in many Gram-negative bacteria. Located on the bacterial surface, these proteins mediate adhesion to host cells during the early stages of infection [36,47]. The HMTp210 protein of *A. paragallinarum* possesses two independent hemagglutination activity domains, which may recognize distinct host receptors. The type 1 hemagglutination activity domain (aa 176–360) is structurally similar to the YadA protein of *Yersinia* spp. This domain features a loop structure rich in proline and charged residues, which is predicted to be responsible for host cell binding. The type 2 domain (aa 1003–1125) shares structural similarity with the UspA1 protein of *Moraxella catarrhalis.* UspA1 is known to interact with host cell surface molecules such as fibronectin via a binding pocket formed by its head–neck–stalk architecture [24]. Collectively, these structural features suggest that the HMTp210 protein may employ mechanisms analogous to those of YadA and UspA1, utilizing its distinct domains to recognize specific receptors on chicken respiratory epithelial cells and thereby determining its tissue-selective tropism [48]. However, this proposed interaction is a prediction based on structural homology and has not been experimentally validated in *A. paragallinarum*. These predictions provide valuable clues for understanding the pathogenesis of *A. paragallinarum*, but they still require direct experimental validation in this bacterium, such as gene knockout, receptor-binding assays, or animal infection models.

Pilin proteins represent another potential factor for adhesion and colonization of *A. paragallinarum*. Genomic analyses have revealed that *A. paragallinarum* carries genes encoding various adhesion factors, among which *hifB* and *fim3* are commonly detected [49]. These genes encode pilus adhesin components and pilus subunit proteins, respectively. These pili may mediate initial bacterial attachment by recognizing specific carbohydrate receptors on the surface of host epithelial cells [50,51]. Liu et al. discovered that expression of the pilin gene *flfA* might be regulated by a phase variation mechanism. Sequencing analysis of its promoter region identified a poly-cytosine repeat tract located between the −10 and −35 elements, with protein expression correlating with the number of repeats [14]. Many bacterial adhesins dynamically modulate their interaction with host receptors through such expression patterns to achieve immune evasion and adapt to different host microenvironments [52,53]. This regulatory mechanism may determine the tissue specificity of *A. paragallinarum*.

### 3.3. Synergistic Pathogenic Mechanisms with Commensal Bacteria

The pathogenicity of *A. paragallinarum* and its tissue tropism for the nasal cavity and the infraorbital sinus are closely linked to the local commensal microbiota of the respiratory tract. *A. paragallinarum* is NAD^+^ (V factor) dependent, yet the NAD^+^ abundance in the healthy chicken respiratory tract is insufficient to support its survival and growth. Through epidemiological investigation and in vitro experiments, Wu et al. found that *Staphylococcus chromogenes* in the chicken respiratory tract plays a synergistic role in *A. paragallinarum* infection. *S. chromogenes* can directly synthesize and secrete NAD^+^. It can also cause membrane damage to mammalian cells, particularly epithelial cells, leading to passive release of intracellular NAD^+^. Additionally, it upregulates the expression of NAMPT, a key enzyme in the host cell NAD^+^ salvage synthesis pathway, thus promoting cellular NAD^+^ synthesis and release. Collectively, these actions elevate local NAD^+^ concentrations in the respiratory tract, creating favorable conditions for *A. paragallinarum* colonization and proliferation. Compared to mono-infection with *A. paragallinarum* alone, co-infection with *S. chromogenes* resulted in aggravated clinical symptoms, an approximately 100-fold increase in bacterial loads in the nasal cavity and infraorbital sinuses, and more severe histopathological damage. Conversely, eradication of *S. chromogenes* using vancomycin halted disease progression caused by *A. paragallinarum* and reduced bacterial loads [18].

Research by Zhu et al. further confirmed the synergistic role of respiratory tract commensal bacteria in the pathogenesis of *A. paragallinarum*. Among commensal bacteria isolated from the respiratory tract of diseased chickens, various Gram-positive species (e.g., *Staphylococcus, Bacillus, Enterococcus*) were found to promote the growth of *A. paragallinarum*. On one hand, commensals provide nutritional support. Some strains exhibit hemolytic activity, lysing erythrocytes to release NAD^+^-containing cellular contents. Additionally, *Bacillus* spp. can directly release NAD^+^, providing the essential growth factor for the pathogen. Moreover, they confer antibiotic protection. When co-cultured with *Bacillus*, *A. paragallinarum* exhibited significantly reduced susceptibility to cefotaxime and ampicillin, with bacterial counts increasing over 1000-fold compared to monoculture. Conversely, *A. paragallinarum* enhances the tolerance of *Bacillus* spp. to doxycycline, potentially via its tetracycline resistance gene *tet (B)*, establishing a bidirectional model against antibiotics. Animal infection models further verified that co-infection induced more severe clinical signs such as facial swelling and nasal inflammation, significantly increased bacterial loads of *A. paragallinarum* in the nasal cavity and infraorbital sinuses, and caused aggravated histopathological damage including submucosal thickening, extensive inflammatory cell infiltration, and destruction of epithelial villus structures [54,55].

However, the growth-promoting effect of commensal bacteria varies among different commensal species and strains. Additionally, host temperature can influence NAD^+^ production from commensals, as Wu et al. [18] showed that *S. chromogenes* produces more NAD+ at 42 °C (chicken body temperature) than at 37 °C. These findings suggest potential variability across host environments [18,54,55].

Taken together, the above studies demonstrate that commensal bacteria promote *A. paragallinarum* infection through multiple mechanisms. At the molecular level, commensals provide NAD^+^ via direct secretion, hemolysis, and upregulation of host NAMPT. At the inter-bacterial interaction level, beyond nutritional support, they confer antibiotic cross-protection, and this mutualism is bidirectional. At the ecological niche level, this growth-promoting capacity is widespread among diverse Gram-positive commensals, representing a community-level phenomenon rather than isolated cases. Current research in this area has largely focused on promoting pathogen survival and antibiotic resistance, with limited direct investigation into host immune regulation. In addition, existing studies have primarily relied on classical methods; future application of metagenomics, dual RNA-seq, and spatially resolved metabolomics will help provide a comprehensive understanding of the metabolic network of the respiratory microbiota and its immunomodulatory effects.

## 4. Immune Responses Elicited by *A. paragallinarum*

### 4.1. LPS/TLR4-Mediated Innate Immune Response

**Figure 3:**
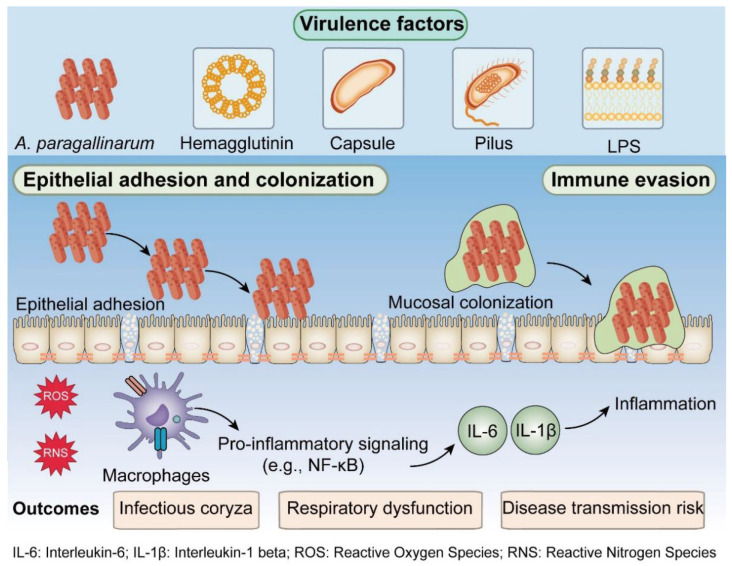
Major virulence factors and host inflammatory responses in *A. paragallinarum* infection. Key bacterial virulence factors, including hemagglutinin, capsule, pilus, and LPS, mediate epithelial adhesion and mucosal colonization. Colonization induces the production of ROS and RNS, which trigger pro-inflammatory signaling (e.g., NF-κB pathway), leading to the secretion of IL-6 and IL-1β and subsequent inflammation. These host responses contribute to clinical outcomes such as infectious coryza, respiratory dysfunction, and increased disease transmission risk. Additionally, immune evasion mechanisms further facilitate bacterial persistence.

Infection with *A. paragallinarum* triggers a local inflammatory response in chickens (Figure 3). Pathological examination of naturally infected chickens revealed that sinus exudates are predominantly consisted of heterophils, and were accompanied by infiltration of histiocytes, lymphocytes, and plasma cells. Foci of mucosal loss and regional edema are also observed. This inflammatory infiltration may extend to adjacent tissues such as the nasal cavity, frontal bone, eyelids, and wattles [30]. Studies have indicated that the lipopolysaccharide (LPS) of *A. paragallinarum* is a key component in activating the host innate immune response [56,57,58].

Research by van den Biggelaar et al. demonstrated that LPS induced the production of nitric oxide (NO), the pro-inflammatory cytokine IL-1β, and the chemokine CXCLi1 in macrophages via the Toll-like receptor 4 (TLR4) pathway [57]. Boucher et al. found that expression of TLR4 and its co-receptor CD14 was significantly upregulated in the nasal tissues of infected chickens. The increased CD14 enhanced the TLR4-mediated recognition of LPS, thereby triggering the MyD88-dependent signaling pathway. This activation led to early nuclear factor-κB (NF-κB) activation, resulting in significant upregulation of pro-inflammatory cytokines such as IL-6 and chemokines like IL-8, which play central roles in recruiting immune cells and initiating the inflammatory response at the site of infection [59,60]. Guo et al. provided further confirmation, showing that at one day post-infection (dpi), gene expression of both TLR4 and its adaptor protein MyD88 was markedly upregulated in the spleen and nasal cavity of chickens. This subsequently triggered a dramatic upregulation of IL-1β and IL-6 (with an approximately 229-fold increase in expression) in local nasal tissues. These findings indicate effective engagement of the TLR4/Myd88 pathway in inducing a host pro-inflammatory response during the early stages of infection [13].

In addition, outer membrane vesicles (OMVs) secreted by *A. paragallinarum* serve as carriers of LPS and contribute to immune activation. Mei et al. found that the endotoxin (LPS) concentration in OMVs purified from *A. paragallinarum* was 211.2 EU/mL. In vitro experiments showed that stimulation of the chicken macrophage cell line HD11 with OMVs significantly upregulated the gene expression of key inflammation-related factors—IL-1β, IL-10, and inducible nitric oxide synthase (iNOS)—producing a response similar to that induced by purified LPS. Regarding humoral immunity, an OMV-based vaccine induced high levels of serum IgG antibodies in SPF chickens, demonstrating its potential to elicit a systemic humoral immune response [4].

In summary, the LPS/TLR4 signaling pathway serves as a central trigger of host defense during *A. paragallinarum* infection, but its excessive activation may exacerbate immunopathology and clinical symptoms. This also suggests that, in vaccine design, the risk of detrimental hyperinflammation caused by residual LPS in whole-cell inactivated vaccines should be carefully considered. Subunit vaccine-based strategies may offer a better balance between protective efficacy and safety by reducing unnecessary inflammatory damage while effectively clearing the pathogen.

### 4.2. Immune Evasion

#### 4.2.1. Adhesin-Mediated Immune Evasion

Adhesins in *A. paragallinarum* not only mediate its colonization of the respiratory epithelium but also facilitate immune evasion. Notably, the key adhesin hemagglutinin (HA) promotes biofilm formation [35,61]. Biofilms provide physical protection for bacteria, enhancing their resistance to both antibiotics and host immune clearance, thereby enabling immune evasion [62]. Furthermore, the type 2 hemagglutination active region (aa 1003–1125) of the HA protein HMTp210 shares high structural similarity with UspA1, an immune evasion protein of *Moraxella* spp. [24]. UspA1 confers resistance to complement killing and facilitates evasion of immune clearance in Moraxella catarrhalis [63]. However, whether the similar structures in *A. paragallinarum* possess similar immune evasion functions has not been experimentally confirmed and warrants further investigation.

#### 4.2.2. MHC-IIlow Monocytes Mediated Immunosuppression

*A. paragallinarum* infection not only triggers local respiratory inflammation but also induces a systemic immunosuppressive state. In an experimental SPF chicken model, Alvarez et al. observed that by day 4 post-infection, the number of circulating monocytes increased significantly. However, the expression of Major histocompatibility complex class II (MHC-II) molecules on these cells was markedly downregulated. In other words, the infection induces a population of monocytes characterized by low MHC-II expression. Although these MHC-IIlow monocytes exhibit high phagocytic activity and participate in pathogen clearance, their downregulated MHC-II results in impaired antigen-presenting capacity. This impairment is associated with reduced proliferation of T cells (particularly CD4^+^ T cells) and diminished antibody production [64]. MHC-IIlow monocytes correlate with adverse clinical outcomes. Among chickens that succumbed to infection, the proportion of the MHC-IIlow monocyte subset in circulating monocytes was abnormally high (>45%), whereas it remained low in infected survivors (<13%) and uninfected controls (<1%). The researchers suggested that this infection-induced “dysfunctional immune response” could be related to the severe clinical signs observed in complicated infectious coryza, which often involves secondary infections [65].

#### 4.2.3. Lipooligosaccharide (LOS) Mediated Complement Resistance

Genomic analyses indicate that *A. paragallinarum* strains carry virulence genes, such as *lpxC*, *manB/yhxB*, and *gmhA/lpcA*, which are involved in LOS and exopolysaccharide biosynthesis [54]. Chen et al. identified that the genome of *A. paragallinarum* contains key heptosyltransferase genes *waaF* and *waaQ* for LOS inner core synthesis, as well as the L6 gene cluster potentially involved in LOS synthesis. Serum bactericidal assays confirmed that an intact LOS structure is critical for the bacterium to resist complement-mediated attack by the host. Compared to the wild-type strain, mutants lacking the key LOS synthesis genes *waaF* or *waaQ* showed significantly decreased resistance to normal chicken serum, with survival rates dropping from 4.89% to 0.0013% and 0.43%, respectively. Additionally, *waaF* and L6 deletion mutants showed attenuated pathogenicity and lower clinical symptom scores in SPF chickens [66]. These findings indicate that LOS enhances bacterial survival and pathogenicity by conferring resistance to complement-mediated killing.

These findings indicate that LOS not only facilitates immune evasion but also represents a potential target for vaccine development. For example, LOS-deficient mutants exhibit attenuated pathogenicity and increased serum susceptibility, making them promising candidates for live attenuated vaccines. Furthermore, conserved LOS epitopes can be used to design subunit or conjugate vaccines that induce antibodies blocking complement resistance, thereby enhancing host clearance. Thus, LOS-based strategies offer multiple avenues for developing safer and more effective vaccines against *A. paragallinarum*.

## 5. Advances in Subunit Vaccines for *A. paragallinarum*

### 5.1. Antigen Selection for Subunit Vaccines Against A. paragallinarum

Current therapeutic strategies against *A. paragallinarum* face challenges. On one hand, clinical isolates have exhibited broad-spectrum resistance to multiple commonly used antibiotics such as enrofloxacin and neomycin, highlighting the importance of prophylactic vaccination [67,68]. On the other hand, widely used whole-cell inactivated vaccines, often formulated in trivalent preparations to cover major serotypes, have limitations. Components like LPS in these vaccines may trigger excessive inflammatory responses [57], and protection is typically serotype-specific, requiring multivalent formulations. Furthermore, these vaccines primarily elicit humoral immunity but induce insufficient local mucosal immunity [69]. Consequently, screening for conserved antigens capable of inducing cross-protection (e.g., outer membrane proteins or common virulence factors) and developing broad-spectrum subunit vaccines based on key protective antigens have become important research directions [70,71].

Compared to whole-cell inactivated vaccines, subunit vaccines contain only specific antigenic components, theoretically offering superior safety profiles. However, their immunogenicity is often weaker. Thus, a core challenge in their design is enhancing immunogenicity to effectively stimulate local mucosal immunity in the respiratory tract [72,73]. Among candidate antigens for *A. paragallinarum*, hemagglutinin (HA) has been confirmed as a key antigenic component for inducing protective immune responses. Immunizing chickens with purified HA antigen effectively protects against challenge infection [16,61,74]. Chen et al. demonstrated that an inactivated vaccine prepared using an HA gene-knockout mutant provided significantly lower protection compared to vaccines containing the intact HA protein [66]. The hemagglutinin protein HMTp210, a surface antigen pivotal in bacterial adhesion and infection, represents a prime target for subunit vaccines [23]. Region 2 of the *HMTp210* gene exhibits high variability, and recombinant proteins that contained the hypervariable region were protected (83–100% protection) against challenge infection with *A. paragallinarum* of the homologous serovar [16]. Lu et al. found that combined immunization with HA and capsular polysaccharide (CPS) provided 80% protection against challenge with serotype B strains. The serum IgG antibody levels were also significantly elevated [45]. Additionally, the vaccines protective efficacy against *A. paragallinarum* positively correlates with hemagglutination inhibition (HI) antibody levels. Booster immunization can significantly increase HI antibody titers, which enhances clinical protection, resulting in reduced clinical symptoms, diminished pathological lesions, and a marked decrease in nasal bacterial shedding post-challenge [75]. This suggests that an effective subunit vaccine should be capable of inducing high levels of HI antibodies.

Beyond HA, fimbrial proteins have been identified as important virulence factors and potential vaccine antigens for *A. paragallinarum*. Liu et al. expressed the fimbrial protein gene *flfA* from *A. paragallinarum* recombinantly in Escherichia coli, obtaining the recombinant FlfA protein (r-FlfA). They demonstrated its function as both a virulence factor and a vaccine antigen. Immunization of chickens with r-FIfA protein significantly alleviated nasal secretions and facial edema induced by challenge with the homologous virulent strain and reduced clinical symptom scores [14].

Future research should focus on identifying more conserved antigens with cross-protective potential. Optimizing multi-antigen formulations and delivery systems will be crucial for developing subunit vaccines capable of providing broad, durable, and highly efficient protection.

### 5.2. How Does Systemic Immunity Confer Local Protection?

For pathogens that primarily infect the respiratory or digestive tracts, vaccination via non-mucosal routes, such as intramuscular or subcutaneous injection, can activate systemic immunity and thus confer protection at local mucosal sites [76]. Subcutaneous vaccination with multivalent inactivated whole-cell preparations has been shown to establish effective systemic immunity in chickens. Following challenge via the infraorbital sinus or nasal cavity, vaccinated chickens show reduced clinical signs (e.g., facial swelling), reduced severity of histopathological lesions, and a significant decrease in bacterial colonization rates within these sites [5,29,77]. Huberman et al. demonstrated that the systemic immune response induced by subcutaneous vaccination with inactivated *A. paragallinarum* effectively protected local sites, such as the infraorbital sinuses, against infection. Compared to the non-vaccinated control, the vaccinated chickens showed a marked reduction in both the incidence and severity of clinical signs following challenge. Further investigation revealed that vaccination effectively inhibited bacterial migration to and colonization of the contralateral infraorbital sinus following unilateral challenge [78]. These findings indicate that vaccine-elicited systemic immune effectors can be rapidly recruited to the initial site of infection. This recruitment not only facilitates pathogen neutralization and alleviates inflammatory damage at the primary focus but also enhances local immune clearance, thereby preventing the spread of infection to adjacent tissues.

Regarding subunit vaccines, although current studies predominantly employ systemic immunization routes such as intramuscular injection, the induced protective immunity can extend protection against respiratory tract infections. Sakamoto et al. found that intramuscular vaccination with a recombinant fusion peptide vaccine (AC45-1) based on region 2 of HMTp210 led to a significant increase in specific serum IgG levels. Upon intranasal challenge with a virulent strain, the immunized chickens exhibited complete clinical protection, whereas the non-immunized control developed typical rhinitis symptoms [79]. Wu et al. showed that subcutaneous immunization with the recombinant HA hypervariable region protein conferred effective immunoprotection to the upper respiratory tract in chickens following intranasal challenge. This demonstrates that systemic humoral immunity elicited by subunit vaccines can effectively defend against local infections at the respiratory mucosa.

Mechanistically, vaccine antigens activate naïve T and B lymphocytes in the draining lymph nodes, inducing high levels of circulating IgG antibodies. A subset of these activated lymphocytes expresses specific homing receptors, such as chemokine receptors and the α4β7 integrin. These cells then migrate via the bloodstream to distant mucosal tissues like the respiratory tract. Within the lamina propria of the mucosa, they differentiate into plasma cells producing secretory IgA (sIgA) antibodies, or establish resident memory T cell and plasma cell populations. This process establishes the first line of immune defense at the portal of entry [80,81,82]. Concurrently, circulating IgG antibodies induced by vaccination can reach the mucosal interstitium of the respiratory tract via vascular leakage or active transport, where they neutralize invading pathogens [83,84,85]. This process is often facilitated by increased vascular permeability associated with local inflammation. This mechanism, whereby systemically induced immunity exerts its effects locally, underlies the protection provided by many vaccines [86,87]. However, the above discussion of lymphocyte homing and IgG transudation is inferred from other mucosal immune systems. The precise mechanisms by which systemic immunity confers local protection in the context of *A. paragallinarum* infection and immunization have not been directly demonstrated and remain to be experimentally elucidated.

### 5.3. Proposed Optimization Strategies

#### 5.3.1. Adjuvants

**Figure 4:**
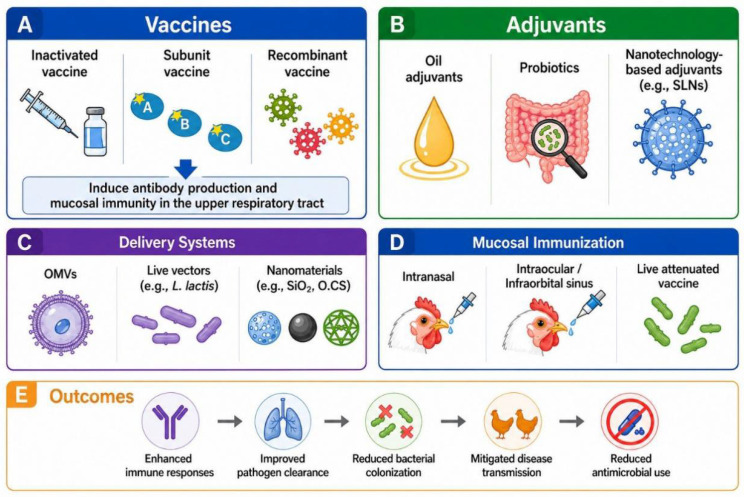
Simplified overview of current immunization strategies against *A. paragallinarum* infection in poultry. (**A**) Main vaccine platforms, including inactivated, subunit, and recombinant vaccines, designed to induce antibody production and respiratory mucosal immunity. (**B**) Representative adjuvants, such as oil adjuvants, probiotics, and nanotechnology-based adjuvants, which enhance antigen stability and immune responses. (**C**) Advanced delivery systems, including outer membrane vesicles (OMVs), live vectors, and nanomaterials, used to improve antigen presentation and vaccine efficacy. (**D**) Mucosal immunization approaches, including intranasal, intraocular/infraorbital sinus administration, and live attenuated vaccines, which provide effective local and systemic protection. (**E**) Expected outcomes include enhanced immune responses, improved pathogen clearance, reduced bacterial colonization, decreased disease transmission, and reduced antimicrobial use in poultry production.

To improve the immunogenicity of *A. paragallinarum* subunit vaccines, particularly their capacity to induce local mucosal immune protection, a synergistic optimization of adjuvants, delivery systems, and vaccination strategies is necessary (Figure 4) [88,89,90].

To overcome the inherent limitation of subunit vaccines in eliciting local mucosal immunity, novel adjuvants have shown promising potential. Among these, probiotics have been validated as biological immunopotentiators capable of effectively enhancing vaccine efficacy [91,92]. Studies indicate that dietary supplementation with *Enterococcus faecium* (*E. faecium*) TC3 alongside *A. paragallinarum* vaccination significantly elevates serum levels of specific antibodies against serotype A and C pathogens. Probiotic adjuvants can elevate IL-12 and IFN-γ levels, thereby promoting Th1-type immune responses; reduce IL-1β and endotoxin levels, mitigating systemic inflammation; and increase glutathione peroxidase (GSH-Px) activity, thereby enhancing the host’s antioxidative status. Challenge experiments further confirmed that this strategy enhances vaccine efficacy and reduces morbidity [26].

Furthermore, drawing on established nanotechnological approaches, the development of novel adjuvants analogous to squalene-based solid lipid nanoparticles (SLNs) holds considerable promise. These formulations exhibit stable physicochemical properties, tolerance to steam sterilization, and suitability for lyophilized storage. Their nanoscale size facilitates more efficient uptake by antigen-presenting cells (APCs), thereby enhancing immunogenicity. In murine models, these adjuvants can induce high-level specific antibodies responses with efficacy comparable in potency to those elicited by Freund’s adjuvant and commercial squalene-based nanoemulsion adjuvants [15].

#### 5.3.2. Vaccine Delivery Systems

Regarding delivery systems, outer membrane vesicles (OMVs) represent a promising antigen display platform due to their immunogenicity, safety profile, and amenability to engineering modifications [93,94]. They can be engineered to display various recombinant antigens, including trimeric autotransporter adhesins (TAAs) [47,95]. For instance, this approach has been successfully applied to deliver the NhhA antigen of *Neisseria meningitidis*, significantly enhancing its immunogenicity [96,97,98,99]. These OMVs released by *A. paragallinarum* are nanoscale antigen delivery systems that carry multiple antigenic components such as lipopolysaccharide and membrane proteins. They can be internalized by immune cells, effectively promoting antigen presentation and immune activation [100]. Evidence indicates that OMVs, administered as a vaccine, can induce high levels of specific IgG antibodies in chickens. They confer approximately 70% protection against challenge with the homologous serotype and exhibit cross-protective effects against heterologous serotypes, significantly alleviating clinical symptoms [4,58]. These findings suggest that employing OMVs as carriers to deliver more immunogenic *A. paragallinarum* antigenic components holds promise for further improving vaccine protective efficacy.

Delivery systems based on live vectors constitute another important avenue for developmen [101]. Mei et al. utilized the food-grade *Lactococcus lactis* (*L. lactis*) strain as a delivery vehicle to express p9, an immunodominant fragment of the outer membrane protein HMTp210. Immunization with inactivated recombinant bacteria not only elicited high levels of serum IgG but also induced the production of nasal mucosal sIgA antibody, conferring 60–80% cross-protection against challenge with serovar A, B, and C strains [102].

In addition, nanomaterials have demonstrated significant value in antigen delivery. Ibrahim et al. evaluated the delivery efficacy of materials such as SiO_2_, Fe_3_O_4_, and oleoyl chitosan (O.CS). These materials not only effectively encapsulate and deliver antigens but also function as adjuvants, significantly enhancing the immune response [3,103,104].

It should be noted that the long-term safety of these novel adjuvants and delivery systems in poultry remains largely unknown, and longer and more in-depth bird health studies are still needed [105]. Direct experimental evidence has demonstrated that certain nanoparticles can produce dose-dependent adverse effects in poultry: dietary supplementation of SiO_2_@AgNPs at 8 mg/kg or above resulted in dose-dependent proinflammatory and immunosuppressive effects, with lesion severity positively correlated with the level of supplementation [106]. Regarding live vector delivery systems, their impact on the host gut microbiota also warrants attention. A poultry study has confirmed that oral administration of recombinant *L. lactis* can alter the cecal microbiota composition in chickens [107]. Although the observed changes were beneficial, this finding indicates that the modulatory effect of live vectors on the gut microbiota is a real phenomenon, and its direction and consequences may vary depending on the vector strain and host background, thus meriting evaluation at an early stage of development. Taken together, these findings suggest that safety indicators such as histopathology, immunoglobulins, and cytokines should be incorporated into the development of novel adjuvants and delivery systems for *A. paragallinarum* vaccines.

#### 5.3.3. Mucosal Immunization

Administration via mucosal routes such as intranasal and intraocular delivery can mimic the natural infection process and effectively elicit local and systemic immune responses. Studies have shown that intranasal vaccination with nano-adjuvanted inactivated vaccines against *A. paragallinarum*, while initiating immunity locally at the mucosa, also induces substantial systemic immune responses. Intranasal immunization with SiO_2_ nanoparticles as an adjuvant induced high levels of serum hemagglutination inhibition (HI) antibodies. This approach elicited 80% to 90% protection against challenge with serovars A, B, and C [3]. Intranasal and intraocular administration of a live attenuated *A. paragallinarum* vaccine achieved 90% clinical protection and significantly reduced bacterial shedding from the nasal cavity of challenged chickens [11]. Moreover, Lu et al. demonstrated distinct advantages of mucosal immunization with a subunit vaccine combining capsular polysaccharide (CPS) and hemagglutinin (HA) protein. Their experiments showed that combined immunization with CPS and HA protein via the infraorbital sinus conferred 80% protection, surpassing the 60% protection achieved through intramuscular immunization. This indicates the critical role of mucosal vaccination in eliciting effective local immunity. The research further noted that while infraorbital sinus immunization did not induce the highest serum antibody levels, it yielded the optimal protective outcome [45]. This suggests that its protective mechanism may be closely associated with the direct elicitation of mucosal immunity with the respiratory tract.

Despite these promising results, mucosal immunization via intranasal, intraocular, or infraorbital sinus routes faces several practical challenges. Effective vaccination requires specialized devices such as droppers for individual bird handling, and the liquid may overflow leading to inaccurate dosing. Compared with mass-application methods such as drinking water, injection, or coarse spray vaccination, these routes are more labor-intensive and time-consuming, limiting their feasibility for large flocks. Furthermore, mucosal vaccine delivery must overcome the mucus barrier, enter target cells, and resist enzymatic degradation, raising new demands for formulation and delivery system development. Regarding safety, some adjuvants may cause local allergic or inflammatory responses at mucosal sites. Therefore, while mucosal immunization offers superior local protection, its practical application will rely on user-friendly delivery devices, cost-effective formulations, and rigorous field validation to overcome these challenges. A summary of the key vaccine strategies discussed in this section, including their protective efficacy, main advantages, and limitations, is provided in Table 1.

## 6. Conclusions

The pathogenic process of *A. paragallinarum* illustrates the intricate interplay between the pathogen, the host, and the local microenvironment. Its specific colonization in the upper respiratory tract relies not only on the recognition of host receptors by key adhesins such as HMTp210 but also on growth support, including factors like NAD^+^ provided by commensal microbiota. While current inactivated and subunit vaccines confer protection by inducing systemic immunity, they are limited in their ability to induce mucosal immunity and cross-protection.

Future research needs to be advanced and integrated across multiple levels. Regarding the mechanisms, further efforts should elucidate the molecular basis of *A. paragallinarum* tissue specificity, particularly the interaction between the pathogen’s adhesins and specific receptors on respiratory epithelial cells, as well as the signaling pathways involved in commensal bacteria-facilitated pathogenesis. Such insights could reveal critical targets for blocking initial infection. In vaccine development, screening for conserved protective antigens facilitated by genomic and proteomic technologies, along with the design of multivalent or chimeric antigens, represents a pivotal approach to overcoming serotype limitations and achieving broad-spectrum protection. Concurrently, the utilization of novel adjuvants (e.g., nanoparticles, probiotics), biomimetic delivery systems (e.g., engineered OMVs, live vectors), and mucosal immunization routes (e.g., intranasal administration) holds significant promise. These strategies are anticipated to enhance the capacity of vaccines to establish a local immune barrier at the respiratory portal, thereby offering a novel approach for the development of subunit vaccines. It should also be noted that although this review focuses on fundamental mechanisms, scaling up production, economic feasibility, regulatory approval, and field application in commercial poultry farms are critical barriers that must be addressed before novel vaccines can be implemented.

## Figures and Tables

**Table 1:** Summary of Vaccine Strategies Against *A. paragallinarum*.

Vaccine Type	Strategies	Key Antigen/Strain	Protective Efficacy	Advantages	Disadvantages/Limitations
Conventional Inactivated Vaccine	Monovalent or multivalent vaccine	Whole bacterial cells (mainly serovars A, B, C)	Good protective efficacy against homologous serovars; significantly reduces clinical morbidity and lesion severity	Mature technology; induces strong systemic humoral immunity	Strong serovar specificity; LPS and other components may cause excessive inflammatory responses; insufficient mucosal immunity
Subunit Vaccine	Recombinant protein	HA protein	83–100% protection rate against homologous serovars	Well-defined components; high safety; induces high HI antibody titers; high expression in *Escherichia coli* (*E. coli*) with low production cost	High variability in hypervariable region requires consideration in vaccine design; expression and purification difficulties for some recombinant proteins
	Recombinant protein	Fimbrial protein FlfA	Significantly reduces clinical symptoms after homologous challenge	Small protein size; easy expression and purification in *E. coli*; targets conserved adhesion factors; high safety	Not expressed in some field strains; heterologous protective efficacy unknown
	Combined antigen	CPS + HA protein	80% homologous protection rate; combined immunization better than single antigen	Well-defined components; high safety	Heterologous protective efficacy unknown
Improved Vaccine	Probiotic immune enhancer	Bivalent inactivated oil-adjuvanted vaccine + *E. faecium*	Low protection rate with vaccine alone; addition of *E. faecium* increases protection by 7% and reduces morbidity by 25–34%	Significantly increases antibody levels; reduces bacterial load	Heterologous protective efficacy unknown
	Live vector delivery	*L. lactis* delivering p9 fragment of HMTp210 protein	60–80% cross-protection against challenge with serovars A, B, and C	Food-grade safe vector; induces both serum IgG and mucosal sIgA; good cross-protection	Requires Nisin induction and relatively complex preparation process
	Probiotic immune enhancer	Bivalent inactivated oil-adjuvanted vaccine + *E. faecium*	Low protection rate with vaccine alone; addition of *E. faecium* increases protection by 7% and reduces morbidity by 25–34%	Significantly increases antibody levels; reduces bacterial load	Heterologous protective efficacy unknown

Note: Other vaccine improvement strategies mentioned in the literature lack research data on *A. paragallinarum* and are therefore not listed in this table.

## Data Availability

No new data were created or analyzed in this study. Data sharing is not applicable to this article.
